# Items

**Published:** 1872-02

**Authors:** 


### ITEMS.

THE Boston Medical & Surgical Journal enters upon its eighty-sixth volume, with Dr. F. W. Draper as assistant editor.

THE Chicago Medical Examiner, edited by Prof. N. S. Davis & Son—the former one of the best informed physicians of this country—puts on a new and improved appearance. It will be issued twice a month, instead of monthly, as heretofore.

The Cincinnati Medical Repertory has been changed to The Cincinnati Medical News, edited by J. A. Thacker, M. D., and Drs. Brumble, Miles and Reed as associate editors.

The Clinic, is the title of of a new weekly medical journal published at Cincinnati, O. It is devoted, in large degree, to foreign medical literature, and is valuable, particularly, on this account. It is edited by Jas. T. Whittaker, M. D., with Drs. Dawson, Conner, Sealy, Kearnes, Reamy, Palmer, Nickles, Cleaveland and Bartholow as associate editors.

Dr. S. W. Butler, of Philadelphia, in the Medical & Surgical Reporter, announces that he will shortly issue "Dr. J. M. Toner's Medical Register & Directory of the United States." Circulars are being sent to over 50,000 physicians, asking information. We hope, with Dr. Butler, that these Circulars will be promptly responded to. The work of Dr. Toner will be of the greatest advantage to the profession, and should, when published, be in the hands of every physician. It is expected the work will appear about the first of March.

We have received from Dr. J. M. Toner, of Washington City—one of the Corresponding Editors of the Companion—a number of very instructive and interesting diagrams. Diagram No. 1 shows the proportion of males to females of the same age, in the United States., according to the enumeration in the census of 1860. Diagram No. 2 shows the ages at which an excess of one sex over the other exists in the different States and Territories of the United States, from data given in the census report of 1860. This is a very interesting diagram, and very ingenuously and curiously shows the excess of one sex over the other. Diagrum No. 3 shows the proportion of white children of both sexes under fifteen years, to the one thousand white females between fifteen and fifty years, the nubile age in each State at every decade from 1800 to 1870, from data given in the several reports of the United States census, to illustrate, graphically, the fact of the decline of the birth-rate in the United States. Diagram No. 7 shows the aggregate mortality in the United States, by age and sex, constructed from 392,821 deaths returned and recorded, for the year ending June 1st, 1860, in the census of that year.

Dr. P. O. Hooper, President of the Arkansas State Medical Association, in his address to that body, remarked :

"We throw out, as a suggestion worthy of consideration, that at stated periods reports be made of the climatology and diseases of each section of the country, and that these be consolidated and published under the direction of the surgeon-general of the army, for the use of scientific medical men ; and that the operations of the "signal corps" be so extended as to embrace every portion of the Union ; that a station be located at the capital of every State and Territory, and at other advisable places, from which shall be sent daily reports by telegraph to the federal capital, of the temperature, weather, currents of wind, ozonic state of the atmosphere, and everything of scientific interest to the physician phyicist. This is an object worthy the attention and undertaking of a great government.

We are gratified to learn that the suggestion of Dr. Hooper has received the endorsement of the signal officer of the Covernment. We endorse the suggestion as worthy the encouragement and assistance of the Government, and hope to see his views practically carried into execution before many months.

---

Treatment of Nævus by the Galvanic Cautery.—Dr. Maas, of Breslau, has collected in the *Archiv fur Klinische Chirurgie* (vol. xii.) the histories of 112 cases of nævus treated by the galvanic cautery. The results were as follows : Capillary Nævus—cured, 32 ; improved, 1 ; result unknown, 1. Cavernous or venous nævus—cured 72 ; improved, 8 ; died, 3. Arterial or racemose nævus—cured, 2 ; improved, 1. Nævus combined with other tumours—cured, 6 ; improved, 1 ; result unknown, 2. He derives from the examination of the cases the conclusion that the galvanic cautery is followed by the best results in nævus, and is much safer than the injection of perchloride of iron or any other coagulating fluid. It would, however, be wrong to say positively that the remedy is indicated in all cases of nævus. As Virchow has well remarked, the surgeon must take the circumstances of each case into consideration. The battery used in the cases referred to was that of Middeldopf.—*Brit. Med. Journal*, Sept. 30, 1871.

